# COVID-19 Preparedness and Response: Validation of a Rapid Assessment Tool to Evaluate Priorities of Health Workers at the Grassroots Level

**DOI:** 10.3389/fpubh.2021.562600

**Published:** 2021-06-29

**Authors:** Bach Xuan Tran, Chi Linh Hoang, Nguyen Thao Thi Nguyen, Huong Thi Le, Hai Quang Pham, Men Thi Hoang, Tu Huu Nguyen, Carl A. Latkin, Cyrus S. H. Ho, Roger C. M. Ho

**Affiliations:** ^1^The Department of Health Economic, Institute for Preventive Medicine and Public Health, Hanoi Medical University, Hanoi, Vietnam; ^2^Bloomberg School of Public Health, Johns Hopkins University, Baltimore, MD, United States; ^3^Center of Excellence in Behavioral Medicine, Nguyen Tat Thanh University, Ho Chi Minh City, Vietnam; ^4^Duke University School of Medicine, Duke University, Durham, NC, United States; ^5^Institute for Global Health Innovations, Duy Tan University, Da Nang, Vietnam; ^6^Faculty of Medicine, Duy Tan University, Da Nang, Vietnam; ^7^Faculty of Pharmacy, Duy Tan University, Da Nang, Vietnam; ^8^Vietnam Young Physicians' Association, Hanoi, Vietnam; ^9^Department of Psychological Medicine, National University Hospital, Singapore, Singapore; ^10^Department of Psychological Medicine, Yong Loo Lin School of Medicine, National University of Singapore, Singapore, Singapore; ^11^Institute for Health Innovation and Technology, National University of Singapore, Singapore, Singapore

**Keywords:** COVID-19, setting priority, medical student, health workers, Vietnam

## Abstract

Since the initial phases of the COVID-19 outbreak, international recommendations for disease control have been readily available. However, blind implementation of these recommendations without grassroot-level support could result in public distrust and low adherence. This study evaluated the use of a public health priorities survey to rapidly assess perceptions of local health workers. A cross-sectional study using a web-based survey was conducted among 5,847 health workers and medical students from January to February 2020 to evaluate the level of prioritization of various public health measures. Measures with the highest levels of prioritization were “Early prevention, environmental sanitation, and improvement of population health” and “Mobilization of community participation in disease control,” which were concordant with policies implemented by the Vietnamese government. This study also demonstrated a high level of internal validity among survey items and shared ranking of priorities among all occupational groups. The use of this public health priorities survey was found to be effective in identifying priorities as identified by grassroots health workers to provide real-time feedback to the national government. However, future iterations of this survey should consider limiting the use of each prioritization score to ensure that responses represent the reality of source limitations and consider focusing on medical professionals and community workers due medical students' limited experience with Vietnam's healthcare infrastructure.

## Introduction

On 31 December 2019, the World Health Organization (WHO) China detected a cluster of pneumonia cases of unknown etiology at Wuhan Jinyintan Hospital in Hubei province, China (1). One week later, on 7 January 2020, the virus now called SARS-CoV-2 was isolated (1). On 11 February 2020, the WHO officially named the disease associated with SARS-CoV-2 infection as COVID-19 (2). Though COVID-19 is associated with lower case-fatality rates, it has proven to be more infectious (3) than both SARS-CoV and MERS-CoV, which caused the severe acute respiratory syndrome (SARS) outbreak in 2003 (4) and of Middle East respiratory syndrome (MERS) in 2012, respectively (5, 6). These differences are likely due to variations in viral reproduction rates along with a large number of asymptomatic SARS-CoV-2 cases contributing to high rates of transmission (5, 7). By 7 April 2020, there were 1,279,722 confirmed cases globally, with over 72,600 deaths in 203 countries across the world (8).

Previous studies have argued that the rapid acceleration of the COVID-19 pandemic was a consequence of inadequate preventative measures (9, 10). This pandemic has demonstrated that no matter how robust a country's health system may be, preparedness is crucial to preventing disease spread (11). Indeed, even some of the most developed health systems in the world have faltered due to a lack of early preventative measures (12–14). For middle-income countries such as Vietnam, unpreparedness could result in even worse consequences given the constrained healthcare infrastructure and resource limitations (15). As such, mechanisms for early and efficient public health planning and priority setting are critical to ensure appropriate disease prevention and control (16–19).

Since the initial phases of the COVID-19 outbreak in Hubei province, there has been widespread guidance from health authorities globally regarding disease prevention and control measures. Standard public health interventions, including isolation and quarantine, physical distancing, and community containment measures, were recommended to control disease transmission (20). The WHO also issued guidelines regarding the importance of establishing research priorities, facilitation of clinical trials, and coordination of efforts to contain SARS-CoV-2 spread (21).

Yet, though international recommendations were readily available, blind implementation of these recommendations without grassroots-level support could result in public distrust and low adherence (22). As such, input from local health workers would be helpful in ensuring that public health interventions reflect the specific needs of each community and increase local support. To supplement data-driven disease control measures, this study evaluated the priorities of Vietnamese health workers and medical students regarding public health interventions to prevent SARS-CoV-2 spread in Vietnam. Health workers and medical students were chosen because they are more likely to be knowledgeable about COVID-19 and Vietnam's healthcare infrastructure. Beyond identifying public health priorities by grassroots health workers during the initial phase of COVID-19 in Vietnam, this study aims to validate the use of a public health priorities survey to rapidly assess local needs during times of pandemic.

## Methods

### Study Setting and Participants

Due to its shared border with China, ground zero of the COVID-19 epidemic, Vietnam was at a higher risk of importing positive cases from Hubei province when the pandemic began in January 2020. As such, from January to February 2020, a web-based survey was conducted among 5,847 health workers and medical students in Vietnam to study the perceptions of COVID-19 in the country. Participants were asked to take part in this survey if they met the following criteria: (1) involvement in Vietnam's COVID-19 response either as a medical student, medical professional, or community worker, (2) age 18 years or older, (3) living in Vietnam for at least 6 months, (4) agreement to participate in the study through online informed consent, and (5) ability to read and respond to the questionnaire.

The recruitment process was initially focused on several core groups consisting of individuals from the Vietnam Young Physician Association and medical universities in Hanoi, Da Nang, and Ho Chi Minh City. These groups were selected to reflect diversity in demographics, including age, gender, and occupation. Individuals in these groups were more likely to be well-connected to peers whom they recruited to participate in the study. Using snowball sampling, a total of 5,847 people were recruited through online invitations to take part in this study.

### Measurements and Instruments

General characteristics collected included gender, age, marital status, level of education, and work-related characteristics, which included the administration level, occupation, and workplace location. Participant occupation was designated as either a medical student, medical professional, or community worker—with community workers defined as those working to prevent spread COVID-19 at the local level. Given that Vietnam's health system is divided into the central, provincial, and district/communal level clinics along with academic health centers associated with universities, participants were also asked to report the administration level in which they are employed. Not all participants answered all questions in the survey, so we only include questions with response rates >95%.

The questionnaire was designed based on the rapid response of Vietnamese government on COVID-19. The content not only covers considerable efforts of public health measures, such as quarantine, wearing masks, isolation, enforcing border closure, but also efforts to strengthen the healthcare system, scientific research capacity. Therefore, it can assess the measure of disease control with a multiple perspective. Participants were assessed for their level of prioritization of various public interventions to contain COVID-19 with questions relating to 12 public health measures including environmental sanitation, knowledge enhancement, development of epidemic forecast systems to provide early warning, and coordination of local actors. For the level of prioritization, scores ranged from 0 beings “not important” to 10 being “extremely important.” These items were categorized into two domains: “Intersectoral approaches to disease prevention” and “Systemic approaches to preventative medicine.” Intersectoral approaches were defined as interventions that rely on coordination of various community-level actors, a bottom-up approach, whereas systemic approaches were defined as interventions that rely on governmental-level organization, or top-down. These domains were constructed using the mean score of all items in each domain.

### Statistical Analysis

The collected data were analyzed by using STATA 15.0 (Stata Corp. LP, College Station, TX, USA). Exploratory factor analysis (EFA) was utilized to evaluate the construct validity of the questionnaire. The Horn's parallel analysis was used to define number of factors. An orthogonal varimax rotation with Kaiser normalization was applied for exploring the scale of items to increase the interpretability of study results. The cut-off point for factor loading was defined at a value of 0.46. Confirmatory Factor Analysis (CFA) was applied to calculate some model fit indices (Model chi-square, RMSEA: Root Mean Square Error of Approximation, CFI: Confirmatory factor index, NFI: Normed Fit Index, SRMR: Standardized Root Mean Square Residual). We measured the internal consistency of each factor using Cronbach's alpha.

Descriptive statistics were used to characterize data frequency, percent, mean, and standard deviation. Inferential statistics were applied to compare the three occupational groups by *T*-test or Mann Whitney test for quantitative variables and by the Fisher-exact test or Chi-square test for qualitative variables. A Tobit multivariable regression model was applied to identify factors associated with each type of channel. Poisson regression models were used to examine factors related to information content. To obtain reduced models, the stepwise forward selection was utilized. Only predictors which have log-likelihood ratio test with a *p*-value less than of 0.2 were included in the final model. Statistical significance was defined at a *p*-value of less than 0.05.

## Results

Table 1 shows the basic information of the respondents, including occupation, gender, age, marital status, and area of residence. Participants were classified by occupation as either a medical professional, medical student, or community worker. The majority of the respondents were medical students (89.7%), female (71.9%), living in urban areas (86.6%), and single (92.3%). The mean ages of medical professionals, medical students, and community workers were *M* = 31.9 (SD = 7.8), *M* = 20.5 (SD = 1.7), and *M* = 32.1 (SD = 4.6), respectively. There were significant differences in the level of participation in community activities with medical students demonstrating the lowest levels of participation at 41.5%.

**Table 1:** Demographics of participants by occupation.

	**Medical professionals**		**Medical students**		**Community workers**		**Total**		***p*-value**
	***n***	**%**	***n***	**%**	***n***	**%**	***n***	**%**	
Total	510	8.7	5,247	89.7	90	1.5	5,847	100.0	
**Gender**									
Male	275	54.4	1,306	25.1	52	59.1	1,633	28.1	<0.01
Female	231	45.7	3,903	74.9	36	40.9	4,170	71.9	
**Living area**									
Urban	358	71.2	4,551	88.0	77	87.5	4,986	86.6	<0.01
Rural	145	28.8	619	12.0	11	12.5	775	13.5	
**Marital status**									
Single	190	37.9	5,125	98.6	26	29.2	5,341	92.3	<0.01
Living with spouse	301	60.1	11	0.2	60	67.4	372	6.4	
Others	10	2.0	64	1.2	3	3.4	77	1.3	
**Administration level**									
Central	67	13.3	518	10.1	22	26.2	607	10.6	<0.01
Province	235	46.6	601	11.7	40	47.6	876	15.4	
Below province level	155	30.8	88	1.7	22	26.2	265	4.6	
College/University	47	9.3	3,911	76.4	0	0.0	3,958	69.4	
**Participation in community activities**									
Yes	407	80.6	2,160	41.5	89	100.0	2,656	45.8	<0.01
No	98	19.4	3,041	58.5	0	0.0	3,139	54.2	
**Age group**									
Under 25	62	13.0	4,844	98.7	3	3.5	4,909	89.7	<0.01
25 and above	415	87.0	64	1.3	83	96.5	562	10.3	
	**Mean**	**SD**	**Mean**	**SD**	**Mean**	**SD**	**Mean**	**SD**	***p*****-value**
Age	31.9	7.8	20.5	1.7	32.1	4.6	21.7	4.5	<0.01

As demonstrated in Table 2, the two domains, “Intersectoral approaches to disease prevention” and “Systemic approaches in preventive medicine,” were evaluated by EFA. The Cronbach alpha of the intersectoral approaches and systemic approaches were 0.97 and 0.94, respectively. “Early prevention, environmental sanitation, and population health improvement” had the highest percentage of participants (42.4%) rating it as “extremely important,” whereas “Increasing coordination among local actors” had the lowest percentage of participants (27.2%) rate it as extremely important. The intersectoral and systemic domains had similar mean scores [*M* = 8.0 (SD = 1.8) and *M* = 8.0 (SD = 1.9), respectively]. According to CFA, the model had some acceptable fit indices (CFI = 0.966, NFI = 0.965, SRMR: 0.021).

**Table 2:** Participant satisfaction with and exploratory factor analysis of measures for disease control.

**Items**	**Extremely important priority**		**Intersectoral approaches to disease prevention**	**Systemic approaches in preventive medicine**
	***n***	**%**		
Early prevention, environmental sanitation, and population health improvement	2,480	42.4		0.77
Mobilization of community participation in disease control	2,223	38.4	0.67	
Training on up to date scientific knowledge	2,190	37.7	0.72	
Raising awareness of the impacts of climate change	2,052	35.3	0.65	
Ensuring adequate budget for disease prevention	1,978	34.1		0.67
Periodic surveillance for infectious diseases	1,958	33.6		0.79
Strengthening health communication and education programs	1,853	31.9		0.66
Development of epidemic forecasts systems to provide early warning	1,779	30.6		0.74
Improvement of interdisciplinary scientific research capacity	1,693	29.2	0.77	
Workforce support for preventive medicine sectors	1,688	29.1	0.70	
Development of guidelines for disease prevention	1,662	28.7	0.78	
Increasing coordination among local actors	1,572	27.2	0.80	
Cronbach's alpha			0.97	0.94
Mean			8.0	8.0
SD			1.8	1.9

*Fits for model: Chi-square (p-value) = 3,198.03 (<0.05), RMSEA = 0.102; CFI = 0.966, NFI = 0.965, SRMR: 0.021*.

Table 3 reports the assessment of medical professionals, medical students, and community workers on the importance of various measures in disease control. Overall, the mean score of all measures was 8.0. The highest-scoring measures were “Mobilization of community participation in disease control” *M* = 8.2 (SD = 2.0) in the intersectoral domain and “Early prevention, environmental sanitation, and population health improvement” *M* = 8.2 (SD = 2.1) in the systemic domain. In contrast, lowest-scoring measures were “Improvement of interdisciplinary scientific research capacity” *M* = 7.9 (SD = 2.0) and “Developing systems of epidemic forecasts and early warning” *M* = 7.9 (SD = 2.1) in the intersectoral and systemic domains, respectively.

**Table 3:** Assessing the importance of local disease control measures of health workers and medical students in Vietnam, 2020.

**Assess the importance (range: 0–10)**	**Medical professional**		**Medical students**		**Community workers**		**Total**		***p*-value**
	**Mean**	**SD**	**Mean**	**SD**	**Mean**	**SD**	**Mean**	**SD**	
Intersectoral approaches to disease prevention	8.3	1.7	8.0	1.8	8.2	1.5	8.0	1.8	<0.01^**^
Mobilization of community participation in disease control	8.6	1.9	8.2	2.0	8.6	1.8	8.2	2.0	<0.01^**^
Training to up to date scientific developments	8.5	1.9	8.2	2.0	8.5	1.8	8.2	2.0	<0.01^**^
Raising awareness of the impacts of climate change	8.3	1.9	8.1	2.0	8.4	1.9	8.1	2.0	0.66
Improvement of interdisciplinary scientific research capacity	8.0	2.0	7.9	2.0	8.0	1.7	7.9	2.0	0.06
Workforce support for preventive medicine sectors	8.1	2.0	7.9	2.0	8.0	1.9	7.9	2.0	<0.01^**^
Development of guidelines for disease prevention	8.3	1.9	7.9	2.0	8.1	2.1	7.9	2.0	0.02^*^
Increasing coordination among local actors	8.1	1.9	7.8	2.0	8.0	1.8	7.9	2.0	<0.01^**^
Systemic approaches in preventive medicine	8.4	1.7	8.0	1.9	8.6	1.3	8.0	1.9	<0.01^**^
Early prevention, environmental sanitation, and population health improvement	8.6	1.9	8.2	2.1	9.3	1.2	8.2	2.1	<0.01^**^
Ensuring adequate budget for disease prevention	8.3	2.0	8.0	2.0	8.4	2.0	8.0	2.0	<0.01^**^
Periodic surveillance for infectious diseases	8.3	1.9	7.9	2.1	8.6	1.5	8.0	2.1	<0.01^**^
Strengthening health communication and education programs	8.4	1.9	7.9	2.0	8.5	1.6	8.0	2.0	<0.01^**^
Development of epidemic forecast systems to provide early warning	8.2	2.0	7.8	2.1	8.3	1.9	7.9	2.1	<0.01^**^

* *p < 0.05,*

** *p < 0.01*.

Table 3 also demonstrates significant differences between occupation and level of prioritization across both domains and 10/12 measures, with the exception of “Raising awareness of the impacts of climate change” and “Improvement of interdisciplinary scientific research capacity.” Medical students consistently gave lower prioritization scores in comparison to other occupations on all measures. Nonetheless, despite differences within each measure, when measures are ranked by mean prioritization scores within each occupational group, all three occupations share the same order prioritization for measures within each domain.

Table 4 demonstrates the results of multivariate regression to identify demographic factors associated with disease prevention priorities across intersectoral and systemic domains. Females in this study appeared to give significant higher scores in both domains compared to males (*B* = 0.27; SE = 0.07 and *B* = 0.37; SE = 0.07). Residence in rural areas tend to have lower score (*B* = −0.20; SE = 0.09 and *B* = −0.18; SE = 0.09). Living with a spouse were also positively associated with higher scores in both domains (*B* = 0.81; SE = 0.19 and *B* = 0.97; SE = 0.19). Those 25 years or older gave higher scores in the systemic approach domain in comparison to their counterparts (*B* = 0.50; SE = 0.17; *p* < 0.01). No significant differences between scores were found between the medical professional and community workers vs. medical students.

**Table 4:** Demographic factors associated with disease control priorities among health workers and medical students in Vietnam, 2020.

	**Intersectoral approaches to disease prevention**			**Systemic approaches in preventive medicine**		
	**B**	**95% CI**	**SE**	**B**	**95% CI**	**SE**
Gender (Female vs. male)	0.27^***^	0.14; 0.41	0.07	0.37^***^	0.23; 0.50	0.07
Occupation (Community workers vs. medical professional)	−0.39	−0.90; 0.12	0.26			
Participated in community activities (Yes vs. no)	0.13^**^	0.01; 0.25	0.06	0.12^*^	−0.00; 0.25	0.06
Living area (Rural vs. urban)	−0.20^**^	−0.37; −0.02	0.09	−0.18^**^	−0.36; −0.00	0.09
Marital status (Living with spouse vs. single)	0.81^***^	0.44; 1.18	0.19	0.97^***^	0.59; 1.35	0.19
Age group (> 25 years vs. <25)	0.31^*^	−0.01; 0.64	0.16	0.50^***^	0.18; 0.83	0.17
**Level of health system management**						
College/University (vs. central)	0.28^***^	0.13; 0.43	0.07	0.34^***^	0.19; 0.49	0.08
Below province level (vs. central)	−0.48^***^	−0.80; −0.16	0.16	−0.50^***^	−0.83; −0.17	0.17

*** *p < 0.01,*

** *p < 0.05,*

* *p < 0.1*.

## Discussion

This study implemented a newly developed tool to rapidly assess local disease control priorities of grassroots healthcare workers and medical students. We found a high level of priority across both intersectoral and systemic approach domains. The measures with the highest levels of prioritization were “Early prevention, environmental sanitation, and population health improvement” in the systemic domain and “Mobilization of community participation in disease control” in the intersectoral domain. There was a high level of internal validity of the questionnaire in addition to the shared ranking of public health measures between all three occupational groups despite significant differences in scores within each measure between groups. These significant differences in prioritization scores across both domains and most measures with medical students consistently giving lower scores than other occupations. Important predictors of higher prioritization among participants included a rural residence, age, and participation in community activities.

Given Vietnam's status as a middle-income country and overall scarcity of healthcare resources, preventative health strategies have been critical to contain COVID-19 spread within the country since the outbreak began in Hubei province, China, in early January 2020. Particularly in resource-scarce countries, experts have recommended the early implementation of social distancing and quarantine to ensure that newly diagnosed infections are adequately contained (23). The high levels of prioritization of “Early prevention, environmental sanitation, and population health improvement” echo these recommendations and highlight the healthcare sector's overall support of aggressive early preventative measures to prevent more severe downstream consequences. Moreover, given the limited capacity of Vietnam's healthcare system, participants in this study also demonstrated a high level of prioritization of community mobilization to support disease control measures. Indeed, in previous outbreaks in resource-constrained settings, community-based surveillance and implementation of preventative strategies were crucial in preventing disease spread (24).

In Vietnam, the first COVID-19 case was identified in Ho Chi Minh City on 22 January 2020 (25), and we see the national government's response coinciding with priorities of grassroots health workers, as identified by our study. Shortly after the initial case, the national government implemented measures to restrict SARS-CoV-2 spread through social distancing, quarantine, health status declarations, and improved sanitation (26). At the same time, there was also increasing mobilization of resources to improve local surveillance (26), which allowed for the rapid quarantining and tracing of tens of thousands of individuals (27). Whereas, other countries, such as Korea, initiated mass production of test kits for early detection (28), Vietnam did not have the infrastructure in place to implement widespread testing and as such, relied on social isolation and rapid quarantining of identified cases to reduce dependence on testing. Indeed, through the early collaborative efforts of the government and Vietnamese citizens, the country was able to effectively control the COVID-19 pandemic. For instance, in response to the first case of COVID-19 on January 23, the public have warned to be on the alert for the new infectious disease through reliable channels (i.e., Daily news and up-to-date messages from Ministry of Health) (29). Besides, the journalists also picked up selective terms in order to improve the attitude and the awareness of individuals in fighting the spread of COVID-19 (30). As of 13 April 2020, there were only 266 confirmed cases and zero deaths in the entire country (31).

Beyond highlighting the overall concordance between grassroots health worker and governmental priorities in Vietnam, this study also demonstrates the efficacy of using a public health priorities evaluation to rapidly assess priorities of grassroots health workers across the country, provide the national government with real-time feedback, and ensure health worker participation in national public health planning. In resource-constrained settings, having such a tool allows the central government to allocate resources according to regional needs, as dictated by those directly involved with local public health interventions. This study found a high level of internal validity of the questionnaire within both intersectoral and systemic domains. Further, our data illustrate the shared ranking of public health measures between all three occupational groups, as determined by each group's mean prioritization scores, further highlighting the high level of agreement on public health priorities in this study.

Despite similarities in the ranking of public health measures, when scores within each measure were examined, there were significant differences in mean scores between occupational groups for both domains and most public health measures with medical students consistently giving lower scores than other occupations. This may be due to less experience among medical students with Vietnam's healthcare infrastructure and lower awareness of its limitations—a factor that may also explain why older age was associated with higher prioritization scores. This finding may also be mediated by lower rates of contact with COVID-19 patients among medical students in comparison to medical professionals and community workers. In the first wave of COVID-19, while the public have put on high alert about new infectious disease, this outbreak also accompanied sensemaking (32). Sensemaking is a term that refers to uncertainty surrounding a sudden event and the attempt to find the explanation, and it might decrease the trust on public health measures by ancient phenomenon or cultural deficiency (33). Previous documents had reported related cases regarding infectious disease, for example, AIDS, SARS, avian flu and Ebola (34–37). Thus, our paper suggests further studies to continuously validate the rapid tool in order to evaluate the awareness of public in the later waves of COVID-19. In addition, we see a similar trend when urban vs. rural location was considered in which participants living in urban areas had significantly higher prioritization scores. This is likely related to the higher numbers of confirmed COVID-19 cases in Hanoi and Ho Chi Minh City, resulting in greater levels of interaction with infected patients and/or awareness of nearby infection (31). Though previous reports have indicated that COVID-19 could pose a greater risk to rural areas due to inadequate healthcare facilities (38), it appears from our data that the concrete threat of confirmed COVID-19 cases nearby is associated with higher prioritization of public health measures than the theoretical risk of inadequate healthcare infrastructure in areas low infection rates.

Given these findings, future attempts to use this tool to rapidly assess public health priorities among grassroots health workers could implement some modifications to improve the interpretability of results. First, because healthcare resources are limited, future iterations should limit the number of times in which participants could use each prioritization score—for instance, participants might only be able to assign 10 (“extremely important”) to one public health measure—to ensure that responses represent the reality of source limitations. Second, given medical students' limited experience working within Vietnam's healthcare infrastructure, along with the observed differences in prioritization among medical students vs. medical professionals/community workers, future iterations should consider focusing on medical professionals and community workers.

Limitations of this study included its cross-sectional design, which limits our ability to infer causal relationships. Further, due to the implementation of snowball sampling, the results of this study are not generalizable to the health worker population in Vietnam, though we have attempted to increase external validity with high sample size. In addition, this study implemented self-reporting, which may have introduced recall bias and social desirability bias. We have attempted to avert this by ensuring the confidentiality of all responses.

## Conclusion

This study evaluated the use of a public health priorities survey to rapidly assess priorities of grassroots healthcare workers and medical students during the initial phases of the COVID-19 pandemic in Vietnam. This study highlights a high level of concordance between priorities identified by health workers and those implemented by the government—those measures being “Early prevention, environmental sanitation, and improvement of population health” and “Mobilization of community participation in disease control.” This study also validated the use of our public health priorities survey and found its efficacy in identifying priorities as identified by grassroots health workers to provide real-time feedback to the national government. Future iterations of this survey should consider limiting the use of each prioritization score to ensure that responses represent the reality of source limitations and consider focusing on medical professionals and community workers due to medical students' limited experience with Vietnam's healthcare infrastructure.

## Data Availability Statement

The raw data supporting the conclusions of this article will be made available by the authors, without undue reservation.

## Ethics Statement

The studies involving human participants were reviewed and approved by the Scientific and Ethical Research Committee by Vietnam Youth Research Institute code (DT.KXDTN.19-13).

## Author Contributions

BT, CLH, HP, and NN: conceptualization. BT, CLH, HL, and MH: methodology. BT, CLH, and HP: formal analysis and investigation. BT and CLH: writing – original draft preparation. NN, TN, CL, CSH, and RH: writing – review and editing. All authors contributed to the article and approved the submitted version.

## Conflict of Interest

The authors declare that the research was conducted in the absence of any commercial or financial relationships that could be construed as a potential conflict of interest.
